# Preservation of Fertility in Female Cancer Patients Desiring Future Child Bearing; What is Available and What can be Offered

**DOI:** 10.4021/wjon616w

**Published:** 2013-03-06

**Authors:** Nader Husseinzadeh, Holleh D. Husseinzadeh

**Affiliations:** a Division of Gynecologic Oncology, Department of Obstetrics and Gynecology, University of Cincinnati, 231 Albert Sabin Way. Cincinnati, Ohio 45267, USA; b Taussig Cancer Center, Cleveland Clinic Foundation. 9500 Euclid Avenue, Cleveland, Ohio 44195, USA

**Keywords:** Fertility preservation, Pregnancy, Cancer patients, Invitro fertilization

## Abstract

The aim of this review is to present an overview of available methods for preservation of ovarian function and fertility in female cancer patients who desire to maintain their child-bearing capacity for future pregnancies. A Medline search was conducted. Published articles from American and European studies from 1976 to present were reviewed. The effect of cancer treatment on the ovary, as well as different methods of fertility preservation and their reproductive outcomes are presented. Pregnancy rates vary according to the type of primary malignancy, stage of disease, method of fertility preservation (for example, hormonal therapy, cryopreservation, fertility-sparing surgery), and other confounding factors such as the patient’s age, reproductive capacity, status of partnership, and genetic disposition. The highest rates of successful pregnancy were observed with embryo cryopreservation. Today, higher cure rates and longer survival are a result of earlier cancer diagnosis and treatment. In conjunction with the advances in assisted reproduction, the preservation of ovarian function and fertility is a major part of multidisciplinary care that should be offered to any young female patient with cancer. Fertility preservation in young cancer patients raises a number of ethical issues particularly regarding standard versus experimental therapies, and long-term financial cost.

## Introduction

Overall cancer incidence among women decreased 0.5% per year from 1998 - 2006. Similarly, cancer death rates between 1991 - 2006 decreased by 12.3 % [1]. The reduction in incidence and cancer-related mortality are due to earlier diagnosis and treatment. As a result, the survival rate among young women with malignancies has reached 90-95% [2]. An increasing number of survivors, particularly those younger than 40 years of age, are confronted with the consequences of cancer treatment, namely radiation and chemotherapy, which can have significant impact not only on ovarian function and fertility, but also menopause later in life [3, 4].

Several strategies have been explored for preservation of ovarian function and fertility, although options may be limited for some, depending on both patient-dependent and independent factors, such as regionally available options. In some patients, hormonal therapy and treatment with gonadotropin releasing hormone (GnRH) agonists during chemotherapy has demonstrated ovarian protection by preventing ovulation, yet may be medically-inadvisable in hormone-sensitive cancers. Ovarian stimulation with cryopreservation of oocytes or embryos can be less ideal because of delay in initiation of cancer treatment, but it remains a promising clinical technique with substantial pregnancy rates that continues to improve with advancement of reproductive technology [5]. It is a preferred method in that it permits natural fertility using fresh or frozen and thawed ovarian cortex, but ischemic changes and loss of primordial follicles are two major complications that can occur [6, 7].

In women who are surgical candidates, ovarian transposition may be performed. Auto transplantation can be performed at both orthotopic (within the pelvis) and heterotopic (outside of the pelvis, for example, subcutaneous) sites. The greatest concern with this technique is in seeding the transplantation site with microscopic residual disease within the ovary.

### Effect of radiation on the ovary

Ionizing radiation induces DNA damage in cellular regulatory molecules, resulting in disruption of the normal cell cycle and, ultimately, cell apoptosis. Although radiation has adverse effects on the ovary at any age, the degree of damage to cells depends on the radiation dose, radiation field, and patient age, with older patients being at greater risk of irreversible damage than younger patients. Various reports have been published on the radiation dose necessary to cause loss of ovarian function. Wallace et al [8] reported that application of 14.3Gy to an ovary in a woman over 30 years of age will cause irreversible infertility and (induce early) menopause. Laushbaugh and Casarett [9] reported that women under age 40 are less sensitive to radiation-induced ovarian damage, and an estimated 20Gy is required to produce permanent ovarian failure compared to 6GY, for women over 40 years of age. Chiarelli et al [10] observed that there is clearly a dose- and distribution-dependent relationship between abdominal-pelvic irradiation and the risk of premature ovarian failure. The infertility rate was 22% with radiation doses of 20-35 Gy; in contrast, the infertility rate was 32% with radiation doses greater than 35 Gy.

Although the uterus is relatively resistant to radiation, there is no doubt that radiation can induce irreversible changes in the uterine blood flow and uterine musculature, which can result in hormone-resistant endometrial insufficiency. There is also higher rate of obstetrical complications in patients who received prior pelvic or abdominal irradiation compared to the general population [11, 12]. These complications include spontaneous abortion (38% versus 12%), preterm labor (62% versus 9%), and low birth weight (62% versus 6%). There is no increased risk of teratogenicity, as long as radiation is not administered during pregnancy. Increased incidence of low birth weight babies and spontaneous abortions has been reported, if conception occurred less than a year from the last radiation exposure [13-15].

### Effect of chemotherapeutic agents on the ovary

At birth, a fixed number of primordial follicles are present in the ovary. After puberty, each of these follicles contain a single oocyte arrested in prophase of its first meiotic division and is highly sensitive to cytotoxic or antimetabolite medications. Therefore, exposure to chemotherapy in this phase often leads to cell death [16]. It is important to keep in mind that physiologic follicular depletion is age-related, with the maximum rate of follicle loss occurring around the age of 38 years; at this point, the oocyte reserve is approximately 10% the number present at menarche [17]. Given that many standard chemotherapeutics act by interrupting the cell’s normal proliferative cycle, it is then logical that older women have a higher incidence of ovarian failure and infertility compared to younger women owing to their diminished primordial follicle reserve.

Toxic effects on the ovary additionally depend on the type of chemotherapy and cumulative dose. Cell-cycle nonspecific drugs, such as alkalyting agents, destroy resting primordial cells, whereas antimetabolites like azathioprine or 6-mercaptopurine are cell-cycle specific and spare resting primordial cells, causing less toxicity. Additionally, alkylating agents are known to cause ovarian fibrosis and accelerated follicular oocyte depletion, resulting in a higher ovarian failure rate [18, 19]. Etoposide induces chromosomal damage with frequent aneuploidy and ovulated mouse oocytes [20]. Anthracyclines, vinca alkaloids, and platinum-based chemotherapies have all been shown to induce different types of chromosomal damage in the female murine oocytes, resulting in marked aneuploidy and early embryonic mortality [21-25].

The majority of studies investigating the genetic effects of a single chemotherapeutic agent have been conducted in animals. In clinical practice, however, women are rarely subjected just to a single chemotherapy agent, thus, their cumulative effects can only be inferred. Some commonly-used drug combinations have demonstrated an increased frequency of aneuploidy and abnormal oocyte maturation many years post-treatment [26]. Naturally, large doses of a particular chemical can overwhelm repair mechanism in stem cells, and dose-effect can also explain differences in development of somatic mutations following chemotherapy [27]. These women should try to conceive after a few years of a disease –free interval at least 6-12 months after completion of chemotherapy, due to the possible toxic effect of the treatment on growing oocytes.

## Methods of Fertility Preservation and Results

Though there are several options available, choice of the most desirable method depends on a variety of factors, including the patient’s age, type of the cancer, stage of the disease, time and duration of treatment, and the fertility status of her partner.

### Hormonal therapy

Oral Contraceptive Pills (OCP) which suppresses ovulation have been investigated for protection of ovarian function in women receiving chemotherapy for Hodgkin’s disease. Chapmen et al [28] found more ovarian follicles on ovarian biopsies in 3 of 6 patients taking OCPs, compared to those who were not. In addition, normal menses were re-established in 5 women who discontinued OCP at the end of chemotherapy, and one became pregnant. Whitehead et al [29] found no protective effect from oral contraceptive pills in patients who received chemotherapy. The rate of amenorrhea, oligoamenorrhea, and pregnancy were similar in both groups. Progestational agents (for example, Progesterone (P4) and Medroxyprogestrone acetate) have been investigated on rat and human primordial follicles and were found to be unable to protect ovarian follicles from cytotoxic chemotherapy [19, 30].

### Gonadotropin-releasing hormone (GnRH)

Gonadotropin-releasing hormones have been utilized during chemotherapy to suppress ovarian function and induce medical menopause in order to protect developing ovarian follicles from damage by cytotoxic therapy. There has been some debate about the existence of follicular stimulating hormone (FSH) receptors in primordial follicles and GnRH receptors in the human ovary. Mixed results have been reported [31] regarding the effectiveness of hormonal suppression to protect primordial follicles from damage by cytotoxic agents. Beck-Fruchter et al [32], in a review of the clinical data, reported that among 345 women who received GnRH agonist co-treatment, ovarian function was present in 91% and remaining 9% had premature ovarian failure (POF). In the 234 women who did not receive GnRH agonist co-treatment, ovarian function was present in 41% and failed in 59%. This was in agreement with Blumenthal et al [33] who reported 5% premature ovarian failure in combination GnRH and chemotherapy group versus 55% in the group receiving chemotherapy alone.

There remains concern whether hormonal therapy administered for ovarian protection may increase risk of recurrence in hormone-sensitive cancers. In a recent meta-analysis of 11,906 premenopausal women with early breast cancer, a GnRH-agonist was used as the only systemic adjuvant treatment and did not significantly reduce recurrence or death after recurrence in estrogen-receptor positive breast cancer. The addition of a GnRH agonist to tamoxifen, chemotherapy, or both in fact reduced recurrence by 12.7% and death after recurrence by 15.1% [34]. Studies with tamoxifen demonstrated that its short-term use does not adversely affect oocyte and embryo development [35, 36].

Aromatase inhibitors have been tested as ovulation induction agents as well. Their advantage in breast cancer patients is that peak estradiol levels are lower than those with conventional stimulation regimens [37]. There are relatively new ovarian stimulation protocols using tamoxifen and letrozole that potentially can increase the margin of safety in these patients. When and if a breast cancer patient does not have sufficient time to undergo ovarian stimulation, ovarian cryopreservation can be offered as the last resort [38].

The American Society of Clinical Oncology [39] reports that there is insufficient long-term data regarding the safety and effectiveness of GnRH-agonist and other means of ovarian suppression on female fertility preservation at this time. The panel recommends that patients seeking fertility preservation in the context of cancer treatment be encouraged to enroll in clinical trials.

### Oocyte cryopreservation

Ovarian stimulation with cryopreservation of harvested mature and immature oocytes is another option for women who desire future fertility. Early results with this method were disappointing due to low oocyte survival and fertilization rates. In addition, pregnancy rates after transfer and implantation of thawed oocytes were quite low [40]. In the oocyte, the degree of freezing and thawing damage differ according to the stage of maturation and quality of oocyte and cryopreservation methods [41]. Sonmezer et al [42] reviewed data from 21 studies and found a mean oocyte survival rate of 47%, a mean fertilization rate of 52.5%, and a mean pregnancy rate per thawed oocyte of 1.5%.

However, recent advances in freezing and thawing techniques have resulted in improved survival rate, i.e. intracytoplasmic sperm injection (ICSI), which overcome zona hardening [43], a frequent cause of implantation failure. An alternative technique is vitrification (ultra-rapid IVF embryo freezing) using high concentration of cryoprotectant without ice formation, which is easier and less expensive. Yoon et al [44] reported a survival rate of 85.1±2.9% (320/364,) a fertilization rate of 74.4±3.5% (168/218), an implantation rate of 14.2% (17/120), and a pregnancy rate of 43.3% (13/30), with vitrification using slush nitrogen.

### Embryo cryopreservation

Embryo cryopreservation is one of the most widely available options for preservation of fertility, but there are several limitations to this method. First, the patient requires a male partner or sperm donor. Second, ovarian stimulation prior to oocyte retrieval requiring exposure to large doses of estrogen with the use of fertility drugs may be contraindicated in patients with estrogen-sensitive cancer Third, delay in the initiation of cancer treatment to allow harvesting methods may not be acceptable in some patients [45]. Recent studies show successful ovarian stimulation using letrozole and gonadotropins which is associated with reduced estrogen exposure in patients with endometrial and breast cancer [46]. Oktay et al [47] reported the result of a prospective controlled study comparing ovarian stimulation with tamoxifen and letrozole in breast cancer patients who wished to preserve their fertility via embryo cryopreservation before chemotherapy. In that study, IVF was performed with letrozole (Letrozole-IVF), tamoxifen (Tam-IVF), and a combination of low-dose FSH and tamoxifen (Tam FSH-IVF). Compared with Tam-IVF, both Tam FSH-IVF and Letrozole-IVF patients had greater numbers of ovarian follicles, mature oocytes, and embryos. Peak estradiol (E_2_) levels were lower with Letrozole-IVF and Tam-IVF compared with Tam FSH-IVF. Cancer recurrence rate was similar among all groups and did not appear to be increased, regardless of cancer stage. The study concluded that the Letrozole-IVF protocol was preferable for ovarian stimulation in those with breast and endometrial cancers because of lower peak estrogen (E2) levels.

### Cryopreservation of ovarian tissue

Ovarian cortical tissue contains a large number of primordial follicles that can be cryopreserved in situ and has the advantage of not requiring ovarian stimulation or delaying the initiation of cancer treatment. Moreover, primordial follicles are significantly less susceptible to thermal injury because of their small size, slower metabolic rate, and the absence of zona pellucida. At present, cryopreservation of ovarian tissue is a promising option for the female cancer patient with a realistic chance of fertility preservation. The cryopreservation of ovarian cortical strips has emerged in recent years as an easy, fast, and inexpensive technique [48] and is recommended for prepubertal and premenarcheal patients receiving chemotherapy or pelvic radiation [49].

Orthotopic and heterotropic ovarian transplantation is an additional option in the hands of an experienced surgeon. A major concern about ovarian transplantation is the potential risk that ovarian tissue may harbor malignant cells [50]. Natural pregnancy and live birth have been achieved by orthotopic transplantation from both fresh and frozen ovarian tissue where the fallopian tubes are present and patent [51, 52]. As a site of transplantation, peritoneal tissue appears to be superior to subcutaneous tissue due to more effective neovascularization and less follicular loss in the peritoneal tissue. Heterotropic autotransplantation is less invasive; it permits easy access to the transplanted tissue for monitoring follicular development in the event of reoperation and costs less than orthotopic transplantation. One major concern of ovarian transplantation is the potential risk that ovarian tissue may harbor malignant cells [53]. Additionally, one fears oocyte quality might be compromised due to temperature differences on those sites that may interfere with follicular development and IVF for pregnancy [54].

## Ethical Issues in Fertility Preservation

Fertility preservation in young cancer patients raises a number of ethical questions for both the oncologist and fertility specialist, particularly regarding standard versus experimental therapies, consent from the underage patient, and long-term financial cost. It is important to emphasize conversations should be held early to discuss impact of patient’s cancer or its treatment on reproductive health, as well as fertility-preservation options. It is important to inform cancer patients about the impact that pregnancy or hormonal therapy could have upon their cancer and possible risk of tumor recurrence. In 2006, the American Society of Clinical Oncology composed guidelines related to this issue in cancer patients [39].

Much attention should be paid to the patient’s quality of life, as well as physical and psychosocial wellbeing in these discussions. In the case of child or adolescent patients, parents can provide consent if the minor demonstrates reasonable understanding and gives their assent. The same procedures apply for experimental therapies within clinical trial, where an Institutional Review Board (IRB) agrees the chance of future reproduction outweighs the burden of the procedure.

Patients or their legal guardians also need to provide direction for disposing the patient’s reproductive materials when the patient’s chances of achieving fertility are futile or in the event of patient’s death. This is an important consideration for families as the court currently rules that children born after posthumous conception or implantation are legal offspring of the deceased person [55]. Another concern of many patients is the risk of congenital anomalies or chromosomal defects resulting from the theoretical mutagenic effect of chemotherapy [56]. At this time, there is little evidence to support any increased risk of genetic imprinting disease or risk of spontaneous cancer in children born following in vitro fertilization and intracytoplasmic sperm injection (ICSI) from these patients [57]. Currently there is convincing evidence that assisted reproductive technology treatment (ART) may increase the risk of chromosomal abnormalities among ICSI pregnancies and low birth weight, preterm delivery among all ART singleton pregnancies [58].

Additionally, some patients with inheritable or family cancer syndromes, (for example, breast, ovarian and colorectal) want to reproduce only if their child would be free from the same risk. For these patients, the preimplantation genetic diagnosis (PGD) technique can be utilized to provide reasonable assurance in minimizing the risk of transmission to their offspring [59]. Even when a patient is interested and motivated to pursue these various options, financial limitation may still present barriers. Many insurance companies including Medicaid currently do not cover fertility-preservation procedures [60]. Families must investigate other programs such as www.fertilehope.org/financial-assista

## Discussion

Over the past decade, much progress has been made in cancer care that has resulted in earlier diagnosis higher cure rates and longer survival of the cancer patient. With recent advances made in reproductive technology and infertility, preservation of ovarian function now has become a major part of multidisciplinary care offered to young female cancer patients who desire future child-bearing capacity. These patients require proper counseling about potential fertility-preservation options and a psychologist should be available for referral to facilitate any complex discussions involving the patient, her partner, and her parents. Currently, several techniques are available for those who wish to preserve their fertility and may have the opportunity to conceive after they have survived their cancer.

In summary, many treatments with GnRH have been utilized to suppress follicular development and thus preserve ovarian function during chemotherapy. In those patients who have identified a male sperm donor or partner, embryo cryopreservation is an ideal option, as it is a clinically well-established procedure with high pregnancy rates. However, this is not possible for patient who requires immediate chemotherapy or when hormonal ovarian stimulation is contraindicated. Cryopreservation of oocytes can be used in single women who can undergo ovarian stimulation cycles; however the effectiveness of this method appears to be low. Finally, for those patients in whom chemotherapy cannot be delayed, ovarian tissue cryopreservation and transplantation is ideal; in this technique, cortical ovarian tissue which contains primordial follicles, (either whole ovary with its pedicle, or isolated follicles) is preserved.

At the time of diagnosis, plans for fertility preservation must be taken into account on individual bases, according to patient’s priorities and cancer treatment strategy. Fertility preservation in patients with hormonal receptor positive disease (for example, breast cancer) who would benefit from systemic therapy requires careful consideration, as tamoxifen and aromatase inhibitors have been used in breast cancer patients. Some have recommended gestational surrogacy to minimize the hormonal exposure during pregnancy or offer fertility- preservation that does not require hormonal exposure.

Oncologists treating young female patients with cancer should be aware of two important issues; first, the effect of cancer treatment on the patient’s fertility and second, the available options to preserve her ovarian function and future pregnancy. There are some challenges that need to be resolved concerning optimal fertility preservation, the option of ovarian tissue retrieval, and the ethical-legal issues surrounding valid consent.

Given the multiple treatment and existing recommendations, it proves important for the oncologist to provide the patient proper anticipatory guidance concerning fertility preservation at the time of diagnosis. With the ethical and legal implications, this discussion and ultimate decision could provide stressful for the patient and family. It is important that the oncologist be sensitive to such issues, provided additional counseling services as needed. It is an exciting time in medicine, as today’s oncologists are able to both cure cancer and offer the opportunity to bear children later in life.
